# The Quality of Life of Older Adults in Rural Eastern India and Its Influencing Factors: A Cross-Sectional Study

**DOI:** 10.7759/cureus.55246

**Published:** 2024-02-29

**Authors:** Annu Antony, Swayam Pragyan Parida, Priyamadhaba Behera, Susanta K Padhy, Vikas Bhatia

**Affiliations:** 1 Community Medicine, Al Azhar Medical College, Kumaramangalam, IND; 2 Community Medicine and Family Medicine, All India Institute of Medical Sciences, Bhubaneswar, IND; 3 Psychiatry, All India Institute of Medical Sciences, Bhubaneswar, IND; 4 Community and Family Medicine, All India Institute of Medical Sciences, Bibinagar, IND

**Keywords:** socioeconomic factors, rural areas, community-dwelling older adults, depression prevention, quality of life (qol)

## Abstract

Aims

This study aimed to assess the quality of life (QoL) of older adults in rural Odisha, India, exploring its multidimensional nature across physical, psychological, social, and environmental domains. The impact of depression and various sociodemographic factors on QoL was also investigated.

Methods

The research was conducted in the Tangi block of Khordha district, Odisha, encompassing 468 older adults. The World Health Organization Quality of Life Brief Version (WHOQOL-BREF) questionnaire, Geriatric Depression Scale (GDS-15), and sociodemographic questionnaire were used in data collection. Sampling employed a multistage approach, with statistical analysis utilizing Statistical Package for the Social Sciences (SPSS) version 20 (IBM SPSS Statistics, Armonk, NY), including t-tests for normally distributed data and the Mann-Whitney U test for non-normally distributed data.

Results

The QoL of older adults in rural Odisha showed variability, with physical and social domains exhibiting relatively positive scores compared to psychological and environmental domains. Depression significantly impacted all QoL dimensions, with the most profound effect observed in global QoL and global health. Sociodemographic factors such as employment, substance use, elder abuse, adverse life events, and poverty were identified as significant determinants of global QoL. Additionally, recreational activity, elder abuse, education, and employment significantly affected all QoL domains.

Conclusions

This study reveals the complex landscape of QoL of older adults in rural Odisha. The findings emphasize the need for comprehensive interventions targeting mental health, social support, and environmental conditions to enhance the overall well-being of this population. Policymakers and healthcare professionals should consider these multidimensional factors to develop effective strategies for improving the QoL of older adults in similar contexts.

## Introduction

As the world's population ages, ensuring the quality of life of older adults has become a major concern globally and nationally. Quality of life (QoL) is a complex concept encompassing physical, psychological, environmental, and social well-being, and it is essential to promote the health and well-being of older adults. In recent years, research has focused on examining the quality of life of older adults, identifying factors that affect it, and developing interventions to improve it.

Globally, the United Nations estimates that the number of people aged 60 years and older will more than double by 2050, reaching 2.1 billion [1]. This demographic shift will have significant societal implications in healthcare, social welfare, and the economy [2]. It is, therefore, imperative to understand the quality of life of older adults globally to ensure that their needs are met and that they can lead fulfilling lives in their later years.

Older adults have consistently been shown to have a poor quality of life compared to other age groups. A study by Subramanian et al. (2016) compared the QoL of different age groups in India using data from the World Health Organization (WHO) Study on Global Ageing and Adult Health. The study found that QoL was highest among young adults (age 18-29) and declined gradually with increasing age, with the lowest QoL reported among older adults (age 60 and above) [3]. This study suggests that QoL is declining in older adults, and this requires further understanding.

The stratified healthcare resulted in better access to healthcare, social services, and other resources in urban areas than in rural areas. This has led to better overall QoL among older adults living in urban areas than their rural counterparts [4]. To identify the QoL of older adults in rural areas, we should explore the strong social support networks and cultural traditions in rural areas. Studying the determinants of QoL in rural India can help identify the specific factors contributing to positive or negative QoL outcomes for older adults in these areas. This information can be used to develop targeted interventions and policies to improve the QoL of older adults in rural areas, taking into account the unique cultural and social context of these communities.

The National Programme for Health Care of the Elderly (NPHCE) is a government initiative in India that focuses on improving the health and well-being of older adults in the country. Along with providing comprehensive healthcare for older adults, NPHCE envisions creating a new "architecture for aging" and promoting active and healthy aging through a "society for all ages" [5]. Understanding the factors affecting the QoL of older adults is essential to achieve the vision of NPHCE. In our study, we have assessed the determinants of the quality of life of older adults and their impact on mental health using the World Health Organization Quality of Life Brief Version (WHOQOL-BREF) questionnaire.

## Materials and methods

**Figure 1:**
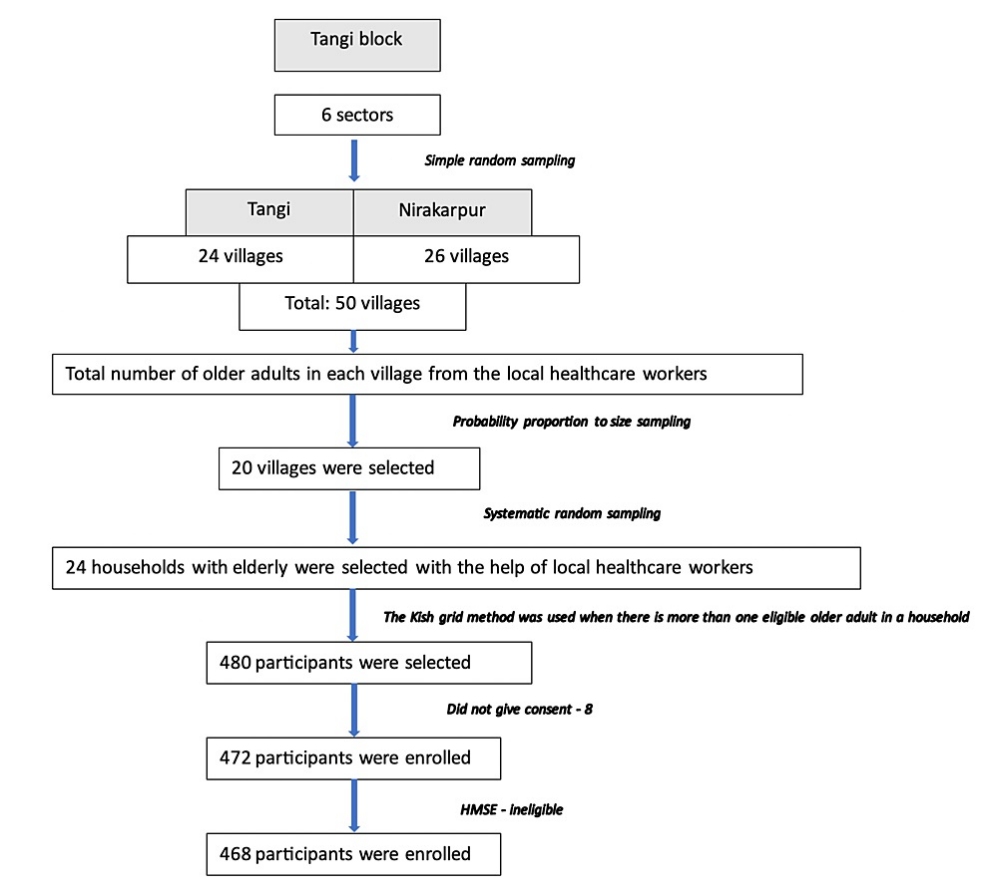
Study flowchart HMSE: Hindi Mental State Examination

The study was conducted among older adults residing in the rural area of Odisha, Eastern India. According to the recent demographic data from records of local governing bodies, the study area, Tangi block of Khordha district, had a 54,387 population with 4,351 older adults. Considering the expected standard deviation (SD) of the QoL score in the elderly population to be 10.21 and a tolerable error of 1% at a 95% confidence interval, the minimum sample size was 400 [6]. Adding a 20% non-response rate, the sample size was calculated as 480. We followed a multistage sampling with probability proportional to size sampling to select the number of households and a Kish grid to select the participant within the household. The sampling strategy is detailed in Figure 1. The study included all individuals who were 60 years old or older and had lived in the area for at least the past six months. However, older adults who had impaired cognition, as determined by the Hindi Mental State Examination (HMSE) by an interviewer, were excluded. The HMSE is an interviewer-applied validated version of the Mini Mental State Examination by Ganguli et al. to be used in the Indian context [7]. Those who were unable to respond due to factors such as hearing loss, inability to speak, lack of comprehension, or illness were also excluded from the study. A total of 472 older adults were selected, and 468 older adults were found to be eligible (Figure 1). All the eligible participants were interviewed using the WHOQOL-BREF questionnaire, Geriatric Depression Scale (GDS-15), and sociodemographic questionnaire.

Outcome variables

The WHOQOL is a 24-item questionnaire in four domains of QoL and global health and global quality of life (Table 1). The domains are as follows: physical domain, psychological domain, social domain, and environmental domain. The physical domain refers to an individual's perception of their physical health, including their pain level, energy, mobility, sleep, and activities of daily living. The psychological domain focuses on an individual's psychological well-being, encompassing their emotional state, self-esteem, body image, cognition, and spirituality. The social domain explores an individual's social relationships, including their social support network, personal relationships, and social inclusion. The environmental domain assesses the individual's physical surroundings, such as access to healthcare, safety, living conditions, transportation, and participation in leisure activities.

**Table 1:** Details of the WHOQOL-BREF questionnaire WHOQOL-BREF: World Health Organization Quality of Life Brief Version

Domain	Total questions	Maximum raw score	Maximum domain score (average *4)	Maximum transformed score ((domain score -4)100/16)
Global health	1	5	20	100
Global quality of life	1	5	20	100
Physical domain	7	35	20	100
Psychological domain	6	30	20	100
Social domain	3	15	20	100
Environmental domain	8	40	20	100

The WHOQOL-BREF, as reflected by its four domains (physical, psychological, social, and environmental domains), is a sound, cross-culturally valid assessment of QoL [8]. The Odia version was validated by Kar et al., with Cronbach's alpha value of 0.81 for the whole scale and that for individual domains were as follows: physical health, 0.71; psychological health, 0.70; social relationships, 0.65; and environmental health, 0.71 [9]. All necessary permissions were taken for using the WHOQOL questionnaire. A total of 36 older adults who did not answer a minimum of 21 (80%) questions were excluded during the analysis of the quality of life. A single interviewer collected the data after a brief training in the Department of Psychiatry, All India Institute of Medical Sciences (AIIMS), Bhubaneswar, and the data was analyzed in means score and standard deviation using Statistical Package for the Social Sciences (SPSS) version 20 (IBM SPSS Statistics, Armonk, NY). Student's t-test was used to compare various determinants of QoL of older adults for normally distributed data and the Mann-Whitney U test for non-normally distributed data. Since the number of data values was more than 25, we used Student's t-test to estimate the distribution of different domains of QoL in various sociodemographic variables [10]. Ethical approval was obtained from the Institutional Ethics Committee of All India Institute of Medical Sciences, Bhubaneswar, as part of the postgraduate thesis protocol with Institutional Ethics Committee (IEC) number IEC/AIIMS BBSR/ PG Thesis/ 2019-20/50.

## Results

The global quality of life of older adults in Eastern India was found to be poor (mean = 46.1) with wide variation among individuals (SD = 27.5). Physical QoL has the highest mean score of 56.1 (SD = 12.8), followed by social QoL with a mean score of 53.6 (SD = 14.8). This suggests that physical health, social relationships, and support are relatively better than other domains of quality of life for older adults in rural Eastern India. The mental health and psychological quality of life are not optimal for older adults in Eastern India (mean = 52.6, SD = 14.8). Also, the physical environment is not very conducive to a good quality of life for the elderly in rural Eastern India (mean = 52.1, SD = 15.1) (Table 2). The wide variation in the SD of global health and global quality of life suggests that the data will be non-parametric.

**Table 2:** Quality of life of older adults living in rural India Data is represented as mean and SD as it was parametric data. QoL: quality of life, SD: standard deviation

Domains	Mean score (maximum score = 100)	SD
Global health	51.9	29.1
Global QoL	46.1	27.5
Physical QoL	56.1	12.8
Psychological QoL	52.6	14.08
Social QoL	53.6	14.8
Environmental QoL	52.1	15.1

The results from our study suggest that, on average, the depressed group scored much lower than the non-depressed group in all types of quality assessed, including global quality, global health, physical QoL, psychological QoL, social QoL, and environmental QoL. The differences in means range from -8.6 for physical quality to -42.9 for global quality (Table 3). Environmental (-12.7) and psychological (-11.9) QoL tend to be the most affected by depression in older adults apart from global QoL (-42.9) and global health (18.7).

**Table 3:** Distribution of QoL score among depressed and non-depressed older adults Data is represented as mean and SD. p-value is considered significant at 0.05. *p < 0.0001 QoL: quality of life, SD: standard deviation

Type of quality assessed (n = 443)	Depressed (n = 252) (mean (SD))	Non-depressed (n = 191) (mean (SD))	Difference
Global quality*	30.6 (18.3)	73.5 (8.1)	-42.9
Global health*	38.03 (21.6)	56.8 (22.2)	-18.7
Physical quality*	51.2 (12.6)	59.8 (11.7)	-8.6
Psychological quality*	45.88 (13.6)	57.8 (12.05)	-11.9
Social quality*	48.3 (15.17)	57.4 (13.5)	-9.0
Environmental quality*	44.9 (15.8)	57.7 (13.3)	-12.7

Significant factors affecting the global QoL of older adults were employment status, substance use, elder abuse, adverse life events, and the poverty line. Being employed had a significantly higher mean global QoL score (mean = 2.79, SD = 0.99) than not being employed (mean = 2.58, SD = 0.87) (p = 0.03). Global QoL was lower in those who reported using substances (mean = 2.56, SD = 0.9) compared to those who did not use substances (mean = 2.76, SD = 0.96) (p = 0.02). Elder abuse survivors had a significantly lower mean global QoL score (mean = 2.26, SD = 0.94) compared to those who did not (mean = 2.72, SD = 0.72) (p = 0.0). Those who reported experiencing adverse life events had a significantly lower mean global QoL score (mean = 2.47, SD = 0.87) compared to those who did not (mean = 2.86, SD = 0.94) (p = 0.0). The standard deviation (SD) varied for each factor, ranging from 0.72 to 1.3. (Appendices). This suggests that the QoL scores for each factor varied widely, indicating the importance of considering individual factors when assessing the QoL of older adults.

The study also found that living with a spouse (mean = 2.9, SD = 0.93) (p = 0.047), being literate (mean = 2.7, SD = 0.9) (p = 0.04), not being abused (mean = 2.6, SD = 0.8) (p = 0.0), not having adverse life events (mean = 2.8, SD = 0.91) (p = 0.0), and not using substances (mean = 2.8, SD = 0.87) (p = 0.03) were significant factors affecting the global health of older adults (Appendices).

Females reported lower physical quality of life scores (mean = 53, SD=11) than males (mean = 56, SD = 12) (p = 0.01). Similarly, illiterate older adults (mean = 52, SD = 12) had lower physical quality of life scores than literate older adults (mean = 56, SD = 11). Employed older adults (mean = 57, SD = 11) reported higher physical quality of life scores than unemployed older adults (mean = 53, SD = 13). Older adults who engaged in recreational activities (mean = 56, SD = 11) and had children living with them (mean = 55, SD = 11) also reported higher physical quality of life scores compared to those who did not engage in recreational activities (mean = 52, SD = 12) and did not have children living with them (mean = 51, SD = 14) (Appendices).

Our study found that education is a significant factor, with literate individuals having a mean score of 48.6 (SD = 12.2) compared to illiterate individuals with a score of 52.2 (SD = 13.7). This difference is statistically significant, with a p-value of 0.004. Employment is another significant factor affecting psychological QoL, with employed individuals having a mean score of 53.3 (SD = 11.5) compared to unemployed individuals with a mean score of 49.8 (SD = 3.9). This difference is statistically significant with a p-value of 0.01. Recreational activity is also a significant factor affecting psychological QoL, with those who engage in recreational activity having a mean score of 58.2 (SD = 13.3) compared to those who do not engage in recreational activity with a mean score of 48.6 (SD = 13.3). This difference is statistically significant, with a p-value of 0.005 (Appendices).

Other factors that have a significant impact on psychological QoL include medical insurance, elder abuse, the presence of children living with older adults, and financial dependence. Those with medical insurance have a mean score of 52 (SD = 13.1) compared to those without medical insurance with a mean score of 49.5 (SD = 13.6), those who have experienced elder abuse have a mean score of 51.5 (SD = 13.1) compared to those who have not with a mean score of 48.3 (SD = 14.3), those with children living with them have a mean score of 51.4 (SD = 13.2) compared to those without children with a mean score of 47.2 (SD = 13.7), and those who are financially dependent have a mean score of 49.2 (SD = 16.6) compared to those who are not financially dependent with a mean score of 52.1 (SD = 16.3). These differences are statistically significant, with p-values of 0.04, 0.008, and 0.03, respectively.

In this comprehensive examination of social quality of life among older adults, our study yielded valuable insights into various determinants. Gender differences, although observed, did not reach statistical significance, with females and males reporting mean social quality of life scores of 51.0 (SD = 14.2) and 53.2 (SD = 14.3), respectively (p = 0.11). Living arrangements proved to be a crucial factor, as those cohabitating with a spouse exhibited a significantly higher mean social quality of life score of 53.2 (SD = 14.2) compared to those without a spouse, who scored 49.4 (SD = 14.4) (p = 0.001). Education played a pivotal role, with literate older adults reporting a mean social quality of life score of 53.5 (SD = 14) in contrast to the score of 49.8 (SD = 14.5) among their illiterate counterparts (p = 0.008). Additionally, engaging in recreational activities positively influenced the social quality of life, with scores of 53.1 (SD = 14.1) for those participating versus 50.4 (SD = 14.6) for those not engaged (p = 0.05). Strikingly, older adults who reported experiencing elder abuse had a significantly lower mean social quality of life score of 49.6 (SD = 16.1) compared to those without such experiences, who scored 52.7 (SD = 13.2) (p = 0.03). These findings underscore the nuanced interplay of sociodemographic factors and interpersonal relationships in shaping the social quality of life of older adults, enriching our understanding of their multifaceted well-being (Appendices).

This study delves into the environmental quality of life among older adults, shedding light on factors that significantly impact their well-being. Gender disparities were not statistically significant, with females and males reporting mean environmental quality of life scores of 50.2 (SD = 15.4) and 50.5 (SD = 15.4), respectively (p = 0.85). Living arrangements emerged as a key determinant, as older adults living with a spouse demonstrated a markedly higher mean environmental quality of life score of 51.4 (SD = 15.0) compared to those without a spouse, scoring 48.0 (SD = 15.9) (p = 0.03). Education played a pivotal role, with literate older adults reporting a mean environmental quality of life score of 52.1 (SD = 14.6), while their illiterate counterparts scored 47.86 (SD = 16.1) (p = 0.04). Employment status proved to be a significant factor, with employed older adults reporting a mean environmental quality of life score of 54.0 (SD = 12.1) compared to the score of 49.1 (SD = 16.2) among the unemployed (p = 0.003). Furthermore, engaging in recreational activities positively influenced environmental quality of life, with scores of 51.5 (SD = 14.4) for participants versus 48.2 (SD = 16.4) for non-participants (p = 0.025) (Appendices).

Table 4 summarizes various factors significantly affecting each domain of quality of life. Employment, elder abuse, and recreational activity have been found to affect all four domains of QoL of older adults significantly. Detailed analysis is presented in the Appendices.

**Table 4:** Summary of factors affecting the QoL of older adults The significance test was conducted using Student's t-test, comparing the mean scores in each domain. p-value is considered significant at 0.05. QoL: quality of life

Variables studied	Global quality	Global health	Physical quality (perception of their physical health, including their level of pain, energy, mobility, sleep, and activities of daily living)	Psychological quality (an individual's psychological well-being, encompassing their emotional state, self-esteem, body image, cognition, and spirituality)	Social quality (an individual's social relationships, including their social support network, personal relationships, and social inclusion)	Environmental quality (an individual's physical surroundings, such as access to healthcare, safety, living conditions, transportation, and participation in leisure activities)
Gender	Non-significant	Non-significant	Significant	Non-significant	Non-significant	Non-significant
Type of family	Non-significant	Non-significant	Non-significant	Non-significant	Non-significant	Non-significant
Living with spouse	Non-significant	Significant	Non-significant	Non-significant	Significant	Non-significant
Education	Non-significant	Significant	Significant	Significant	Significant	Significant
Employment	Significant	Significant	Significant	Significant	Significant	Non-significant
Medical insurance	Non-significant	Non-significant	Non-significant	Significant	Non-significant	Significant
Recreational activity	Non-significant	Non-significant	Significant	Significant	Significant	Non-significant
Elder abuse	Significant	Significant	Significant	Significant	Significant	Significant
Substance use	Significant	Significant	Non-significant	Non-significant	Non-significant	Non-significant
Adverse life events	Significant	Significant	Non-significant	Significant	Non-significant	Non-significant
Disability	Non-significant	Non-significant	Non-significant	Significant	Non-significant	Non-significant
Multiple comorbidities	Non-significant	Non-significant	Non-significant	Non-significant	Non-significant	Non-significant
Any children living with older adult	Non-significant	Non-significant	Significant	Significant	Non-significant	Significant
Financially dependent	Non-significant	Non-significant	Non-significant	Significant	Non-significant	Non-significant
Physically dependent for self-care	Non-significant	Non-significant		Non-significant	Non-significant	Non-significant
Poverty line	Non-significant	Non-significant	Non-significant	Non-significant	Non-significant	Non-significant
Member of organization/group	Non-significant	Non-significant	Non-significant	Non-significant	Non-significant	Significant

## Discussion

The quality of life (QoL) concept aims to encompass holistic well-being, considering both the positive and negative aspects of an individual at a specific point in time [11]. The WHO defines quality of life as "individuals' perceptions of their position in life in the context of the culture and value systems in which they live and in relation to their goals, expectations, standards, and concerns" [12]. However, researchers from diverse disciplines engage in an ongoing debate concerning the definition of QoL of older adults. This discussion intersects with investigations into related concepts such as successful aging, subjective well-being, life satisfaction, and happiness [13]. Even though how to define and measure the QoL in the elderly is still debatable, measuring and understanding factors affecting the quality of life of older adults is imperative to the successful management of the aging population.

Our study explored the QoL of older adults residing in rural areas in physical, environmental, psychological, and social quality domains. We found that physical QoL was better for older adults in rural areas than psychological, social, and environmental QoL. Although global health was satisfactory, the broad SD suggests that individual variations are present [14]. Similar to our finding, Krishnappa et al. compared the QoL of older adults in urban and rural areas and found that physical quality of life was better in rural areas. In contrast, in urban populations, social and environmental QoL was better compared to other domains [4]. It is also noteworthy that depression is associated with a significant reduction in quality of life, consistent in all the domains. Studies by Sahoo et al. [15] and Shah et al. [16] show a high to moderate correlation between depression and the quality of life of the elderly. Although our study could not establish the temporality between depression and quality of life, it is believed that they are correlated and mutually causative [17]. Depressed older adults exhibited a more significant decline in both their environmental and psychological quality of life.

We also explored various factors affecting QoL in different domains. In older adults, recreational activity, elder abuse, education, and employment had significant effects on all the domains, namely, physical, psychological, social, and environmental quality of life. While gender and living with any children had a significant impact only on the physical quality of life, it did not affect the psychological, social, and environmental quality of life of older adults. It is also notable how medical insurance increases the psychological and environmental quality of life of older adults. On the contrary, adverse events in life and disabilities decreased psychological quality of life. Environmental quality of life also improved with being a member of any organization or group.

Musich et al. showed us how employment status has been consistently associated with better health outcomes among older adults, as it provides financial security, access to healthcare, and a sense of purpose [18]. A study from South India comparing urban and rural populations found that employment, education, and thus financial dependency had a significant effect on the urban population rather than on the rural population of older adults [4]. This contrasts our findings as our study revealed that employment and education significantly affect the QoL of rural older adults.

As early as the 1990s, the importance of recreational activities was established [19,20]. However, Rejeski et al. stated that it is time to move beyond primary descriptive associations between recreational activities and work on conceptual frameworks [21]. A similar study also found that recreational activities without vigorous physical activity are more effective in older adults in reducing the risk of depression [22]. An 18-month panel analysis showed that more proximal modifiable outcomes mediate QoL in older adults [23]. Our study also found that while recreational activity affected all domains of QoL, physical activity did not have any significant effect. Further studies on conceptual frameworks with clear definitions of physical activities are required for better understanding.

Elder abuse is defined by the WHO as "a single or repeated act, or the lack of appropriate action, occurring within any relationship in which there is an expectation of trust, where such an act or lack of action causes harm or distress to an older person" [24]. The trauma of elder abuse is well established with low quality of life [25,26]; our study also found that elder abuse significantly reduces quality of life in all domains. In a lifetime abuse study involving 4,467 participants, it was discovered that psychological abuse was linked to decreased autonomy and restrictions in past, present, and future activities. Furthermore, physical abuse causing injuries significantly reduced social participation [26].

Our study was conducted during the immediate post-pandemic period, and the effect of the pandemic on the quality of life is not adjusted in analysis. We employed a robust sample size to enhance the reliability of our conclusions and minimize selection bias through multistage sampling. Standardized assessment tools, such as the WHOQOL-BREF questionnaire and the Geriatric Depression Scale, ensured the reliability and comparability of our results. However, it is important to note that our findings are specific to the rural area in Odisha, limiting their generalizability to other regions, especially urban settings. Furthermore, the cross-sectional nature of our study did not allow for the establishment of temporality, and future longitudinal studies may offer a more dynamic perspective. Additionally, reliance on self-reported data, while practical, may introduce recall bias. Lastly, our study addressed confounders only through probability sampling, without further adjustments during the analysis.

## Conclusions

In summary, our study of older adults in Eastern India revealed a complex landscape of quality of life (QoL). Global QoL was generally poor, with significant variations among individuals. While physical and social QoL showed relatively positive scores, psychological and environmental QoL lagged. Depression had a pervasive negative impact on all QoL dimensions. Various factors, including employment, substance use, elder abuse, adverse life events, and poverty, significantly affected global QoL. Conversely, recreational activity, elder abuse, education, and employment significantly affected all the domains of QoL, namely, physical, psychological, social, and environmental quality of life. This underscores the need for comprehensive interventions addressing mental health, social support, and environmental conditions to enhance the overall well-being of older adults in Eastern India.

## Appendices

Table 5 shows the distribution of factors that affect the global quality of life of older adults.

**Table 5:** Distribution of various factors affecting the global quality of life of older adults Data is represented as mean and SD. p-value is considered significant at 0.05. QoL: quality of life, SD: standard deviation, APL: above poverty line, BPL: below poverty line

Factors		Mean QoL score	SD	p-value
Gender	Female	2.61	0.93	0.55
	Male	2.66	0.91	
Type of family	Nuclear			
	Non-nuclear			
Living with spouse	Yes	2.6	0.93	0.13
	No	2.5	0.9	
Education	Literate	2.6	0.945	0.6
	Illiterate	2.65	0.912	
Employment	Employed	2.79	0.99	0.03
	Unemployed	2.58	0.897	
Medical insurance	Yes	2.69	0.95	0.15
	No	2.57	0.88	
Recreational activity	Yes	2.65	0.92	0.6
	No	2.61	0.927	
Elder abuse	Yes	2.26	0.946	0.0
	No	2.72	0.72	
Substance use	Yes	2.56	0.9	0.02
	No	2.76	0.96	
Adverse life events	Yes	2.47	0.87	0.0
	No	2.86	0.94	
Disability	Yes	2.67	0.9	0.403
	No	2.6	0.94	
Multiple comorbidities	Yes	2.66	0.93	0.068
	No	2.44	0.83	
Any children living with older adult	Yes	2.6	0.83	0.08
	No	2.4	0.94	
Financially dependent	Yes	2.6	0.94	0.35
	No	2.5	0.9	
Physically dependent for self-care	Yes	2.8	1.3	0.4
	No	2.6	0.9	
Poverty line	APL	2.7	0.93	0.15
	BPL	2.5	0.94	
Member of organization/group	Yes	2.6	0.91	0.41
	No	2.6	0.93	

Table 6 shows the distribution of factors that affect the global health of older adults.

**Table 6:** Distribution of various factors affecting the global health of older adults Data is represented as mean and SD. p-value is considered significant at 0.05. SD: standard deviation, APL: above poverty line, BPL: below poverty line

Factors		Mean score	SD	p-value
Gender	Female	2.84	0.9	0.14
	Male	2.96	0.94	
Type of family	Nuclear			
	Non-nuclear			
Living with spouse	Yes	2.9	0.93	0.047
	No	2.7	0.9	
Education	Literate	2.7	0.9	0.04
	Illiterate	2.9	0.94	
Employment	Employed	3.0	0.94	0.3
	Unemployed	2.9	0.92	
Medical insurance	Yes	2.9	0.93	0.1
	No	2.9	0.91	
Recreational activity	Yes	3.0	0.94	0.16
	No	2.8	0.90	
Elder abuse	Yes	3.0	0.93	0.0
	No	2.6	0.8	
Substance use	Yes	3.1	1.0	0.03
	No	2.8	0.87	
Adverse life events	Yes	3.1	0.92	0.0
	No	2.8	0.91	
Disability	Yes	2.8	0.92	0.45
	No	2.9	0.93	
Multiple comorbidities	Yes	2.8	0.90	0.6
	No	2.49	0.93	
Any children living with older adult	Yes	2.93	0.92	0.13
	No	2.75	0.92	
Financially dependent	Yes	2.86	0.91	0.36
	No	2.94	0.93	
Physically dependent for self-care	Yes	3.38	0.74	0.14
	No	2.89	0.92	
Poverty line	APL	2.9	0.9	0.63
	BPL	2.84	0.94	
Member of organization/group	Yes	2.8	0.91	0.51

Table 7 shows the distribution of factors that affect the physical QoL of older adults.

**Table 7:** Distribution of various factors affecting the physical QoL of older adults Data is represented as mean and SD. p-value is considered significant at 0.05. QoL: quality of life, SD: standard deviation, APL: above poverty line, BPL: below poverty line

Factors		Mean score	SD	p-value
Gender	Female	53	11	0.01
	Male	56	12	
Type of family	Nuclear			
	Non-nuclear			
Living with spouse	Yes	55	12	0.09
	No	53	11	
Education	Literate	56	11	0.01
	Illiterate	52	12	
Employment	Employed	57	11	0.007
	Unemployed	53	13	
Medical insurance	Yes	55	12	0.14
	No	53	13	
Recreational activity	Yes	56	11	0.006
	No	52	12	
Elder abuse	Yes	51	13	0.004
	No	55	12	
Substance use	Yes	54.4	13	0.8
	No	54.7	12	
Adverse life events	Yes	54.8	12.4	0.52
	No	54.1	12.1	
Disability	Yes	54.1	11.4	0.58
	No	54.8	13.1	
Multiple comorbidities	Yes	54.1	12.6	0.26
	No	56.2	11.7	
Any children living with older adult	Yes	55	11	0.01
	No	51	14	
Financially dependent	Yes	53	12	0.16
	No	55	13	
Physically dependent for self-care	Yes	58	12	0.37
	No	54	11	
Poverty line	APL	54	11	0.5
	BPL	53	14	
Member of organization/group	Yes	55	11	0.8
	No	53	13	

Table 8 shows the distribution of factors that affect the psychological QoL of older adults.

**Table 8:** Distribution of various factors affecting Psychological QOL of older adults Data is represented as mean and SD. p-value is considered significant at 0.05. QoL: quality of life, SD: standard deviation, APL: above poverty line, BPL: below poverty line

Factors		Mean score	SD	p-value
Gender	Female	50.4	13.2	0.64
	Male	51.1	13.6	
Type of family	Nuclear			
	Non-nuclear			
Living with spouse	Yes	51.4	13.1	0.54
	No	50.7	14.4	
Education	Literate	48.6	12.2	0.004
	Illiterate	52.2	13.7	
Employment	Employed	53.3	11.5	0.01
	Unemployed	49.8	13.9	
Medical insurance	Yes	52	13.1	0.04
	No	49.5	13.6	
Recreational activity	Yes	58.2	13.3	0.005
	No	48.6	13.3	
Elder abuse	Yes	51.5	13.1	0.04
	No	48.3	14.3	
Substance use	Yes	51.0	14.1	0.54
	No	50.1	13.1	
Adverse life events	Yes	51.	13.5	0.6
	No	50.1	13.3	
Disability	Yes	49.3	13.0	0.07
	No	51.6	14.6	
Multiple comorbidities	Yes	51.7	13.4	0.6
	No	51.7	13.7	
Any children living with older adult	Yes	51.4	13.2	0.008
	No	47.2	13.7	
Financially dependent	Yes	49.2	16.6	0.03
	No	52.1	16.3	
Physically dependent for self-care	Yes	47.2	13.7	0.72
	No	50.8	13.4	
Poverty line	APL	51	13.5	0.11
	BPL	48	12	
Member of organization/group	Yes	52.6	13.2	0.07
	No	49.1	13.2	

Table 9 shows the distribution of factors that affect the social quality of life of older adults. 

**Table 9:** Distribution of various factors affecting the social quality of life of older adults Data is represented as mean and SD. p-value is considered significant at 0.05. QoL: quality of life, SD: standard deviation, APL: above poverty line, BPL: below poverty line

Factors		Mean QoL score	SD	p-value
Gender	Female	51.0	14.2	0.11
	Male	53.2	14.3	
Type of family	Nuclear			
	Non-nuclear			
Living with spouse	Yes	53.2	14.2	0.001
	No	49.4	14.4	
Education	Literate	53.5	14	0.008
	Illiterate	49.8	14.5	
Employment	Employed	53.3	13.7	0.2
	Unemployed	51.4	14.2	
Medical insurance	Yes	53.2	14.3	0.08
	No	50.8	14.0	
Recreational activity	Yes	53.1	14.1	0.05
	No	50.4	14.6	
Elder abuse	Yes	49.6	16.1	0.03
	No	52.7	13.2	
Substance use	Yes	51.3	13.2	0.2
	No	53.2	15.1	
Adverse life events	Yes	51.5	14.5	0.43
	No	52.5	17.0	
Disability	Yes	51	13.4	0.21
	No	52.1	14.9	
Multiple comorbidities	Yes	51.9	12.4	0.79
	No	52.1	13.9	
Any children living with older adult	Yes	52.6	14.2	0.055
	No	49.2	15.3	
Financially dependent	Yes	51.8	14.5	0.73
	No	52.3	14.1	
Physically dependent for self-care	Yes	53.1	8	0.8
	No	52	14	
Poverty line	APL	52.2	14	0.45
	BPL	50.5	16.7	
Member of organization/group	Yes	53.4	14.5	0.11
	No	51	14.1	

Table 10 shows the distribution of various factors that affect the environmental quality of life of older adults.

**Table 10:** Distribution of various factors affecting environmental quality of life of older adults Data is represented as mean and SD. p-value is considered significant at 0.05. QoL: quality of life, SD: standard deviation, APL: above poverty line, BPL: below poverty line

Factors		Mean QoL score	SD	p-value
Gender	Female	50.2	15.4	0.85
	Male	50.5	15.4	
Type of family	Nuclear			
	Non-nuclear			
Living with spouse	Yes	51.4	15.0	0.03
	No	48.0	15.9	
Education	Literate	52.1	14.6	0.04
	Illiterate	47.86	16.1	
Employment	Employed	54.0	12.1	0.003
	Unemployed	49.1	16.2	
Medical insurance	Yes	52.2	14.2	0.01
	No	50.8	14.3	
Recreational activity	Yes	51.5	14.4	0.025
	No	48.2	16.4	
Elder abuse	Yes	46.2	0.946	0.0
	No	51.3	0.72	
Substance use	Yes	49.9	15.3	0.45
	No	51.1	15.4	
Adverse life events	Yes	51.4	14	0.17
	No	2.86	0.94	
Disability	Yes	49.7	14.4	0.21
	No	50.6	16	
Multiple comorbidities	Yes	51	14.1	0.26
	No	48.4	16.2	
Any children living with older adult	Yes	46.0	17.05	0.004
	No	51.3	14.8	
Financially dependent	Yes	49.7	15.7	0.28
	No	51.3	14.9	
Physically dependent for self-care	Yes	46.0	17.0	0.4
	No	50.4	15.3	
Poverty line	APL	50.9	14.4	0.09
	BPL	47.4	18.1	
Member of organization/group	Yes	49.0	15.9	0.041
	No	51.9	14.5	

## References

[1] (2024). World population ageing. https://www.un.org/en/development/desa/population/publications/pdf/ageing/WPA2017_Highlights.pdf.

[2] (1988). Social, economic, and demographic changes among the elderly. The aging population in the twenty-first century: statistics for health policy.

[3] Subramanian SV, Subramanyam MA, Selvaraj S, Kawachi I (2009). Are self-reports of health and morbidities in developing countries misleading? Evidence from India. Soc Sci Med.

[4] Krishnappa L, Gadicherla S, Chidambaram P, Murthy NS (2021). Quality of life (QOL) among older persons in an urban and rural area of Bangalore, South India. J Family Med Prim Care.

[5] (2023). National Programme For Health Care Of The Elderly (NPHCE): Operational guidelines. https://main.mohfw.gov.in/sites/default/files/8324324521Operational_Guidelines_NPHCE_final.pdf.

[6] Kumar S G, Majumdar A, G P (2014). Quality of life (QOL) and its associated factors using WHOQOL-BREF among elderly in urban Puducherry, India. J Clin Diagn Res.

[7] Ganguli M, Ratcliff G, Chandra V (1995). Hindi version of the MMSE: the development of a cognitive screening instrument for a largely illiterate rural elderly population in India. Int J Geriatr Psychiatr.

[8] Skevington SM, Lotfy M, O'Connell KA (2004). The World Health Organization’s WHOQOL-BREF quality of life assessment: psychometric properties and results of the international field trial. A report from the WHOQOL group. Qual Life Res.

[9] Kar N, Swain S, Patra S, Kar B (2017). The WHOQOL-BREF: translation and validation of the Odia version in a sample of patients with mental illness. Indian J Soc Psychiatry.

[10] le Cessie S, Goeman JJ, Dekkers OM (2020). Who is afraid of non-normal data? Choosing between parametric and non-parametric tests. Eur J Endocrinol.

[11] Teoli D, Bhardwaj A (2024). Quality of life. https://www.ncbi.nlm.nih.gov/books/NBK536962/.

[12] (2023). WHOQOL: Measuring quality of life. https://www.who.int/tools/whoqol.

[13] Stanley M, Cheek J (2003). Well-being and older people: a review of the literature. Can J Occup Ther.

[14] El Omda S, Sergent SR (2024). Standard deviation. http://www.ncbi.nlm.nih.gov/books/NBK574574/.

[15] Sahoo SS, Kaur V, Panda UK, Nath B, Parija PP, Sahu DP (2022). Depression and quality of life among elderly: comparative cross-sectional study between elderly in community and old age homes in Eastern India. J Educ Health Promot.

[16] Shah F, Solanki P, Tandel N, Valodwala KC (2022). A study to find correlation between depression and quality of life in geriatric population. Int J Sci Healthcare Res.

[17] Huang LT, McMillan SC (2019). Mutual effects of depression on quality of life in patients and family caregivers. Oncol Nurs Forum.

[18] Musich S, Wang SS, Kraemer S, Hawkins K, Wicker E (2018). Purpose in life and positive health outcomes among older adults. Popul Health Manag.

[19] Rejeski WJ, Brawley LR, Shumaker SA (1996). Physical activity and health-related quality of life. Exerc Sport Sci Rev.

[20] Stewart AL, Mills KM, Sepsis PG, King AC, McLellan BY, Roitz K, Ritter PL (1997). Evaluation of CHAMPS, a physical activity promotion program for older adults. Ann Behav Med.

[21] Rejeski WJ, Mihalko SL (2001). Physical activity and quality of life in older adults. J Gerontol A Biol Sci Med Sci.

[22] Antony A, Parida SP, Behera P, Padhy SK (2023). Geriatric depression: prevalence and its associated factors in rural Odisha. Front Public Health.

[23] Phillips SM, Wójcicki TR, McAuley E (2013). Physical activity and quality of life in older adults: an 18-month panel analysis. Qual Life Res.

[24] (2023). Abuse of older people. https://www.who.int/news-room/fact-sheets/detail/abuse-of-older-people.

[25] Fraga S, Soares J, Melchiorre MG (2017). Lifetime abuse and quality of life among older people. Health Soc Work.

[26] Honarvar B, Gheibi Z, Asadollahi A, Bahadori F, Khaksar E, Rabiey Faradonbeh M, Farjami M (2020). The impact of abuse on the quality of life of the elderly: a population-based survey in Iran. J Prev Med Public Health.

